# Biological effects of fulvestrant on estrogen receptor positive human breast cancer: short, medium and long‐term effects based on sequential biopsies

**DOI:** 10.1002/ijc.29682

**Published:** 2015-07-30

**Authors:** Amit Agrawal, John F.R. Robertson, Kwok L. Cheung, Eleanor Gutteridge, Ian O. Ellis, Robert I. Nicholson, Julia M.W. Gee

**Affiliations:** ^1^ Division of Breast Surgery, School of Graduate Entry Medicine and Health, Royal Derby HospitalUniversity of NottinghamDerbyDE22 3DTUnited Kingdom; ^2^ Cambridge Breast UnitCambridge University HospitalsCambridgeCB2 0QQUnited Kingdom; ^3^ University of Nottingham, Nottingham City HospitalNG5 1PBUnited Kingdom; ^4^ Cardiff School of Pharmacy and Pharmaceutical SciencesCardiff UniversityCardiffCF10 3NBUnited Kingdom

**Keywords:** breast cancer, fulvestrant, biology, endocrine resistance

## Abstract

We report the first study of the biological effect of fulvestrant on ER positive clinical breast cancer using sequential biopsies through to progression. Thirty‐two locally/systemically advanced breast cancers treated with first‐line fulvestrant (250 mg/month) were biopsied at therapy initiation, 6 weeks, 6 months and progression and immunohistochemically‐analyzed for Ki67, ER, EGFR and HER2 expression/signaling activity. This series showed good fulvestrant responses (duration of response [DoR] = 25.8 months; clinical benefit = 81%). Ki67 fell (*p* < 0.001) in 79% of tumours by 6 months and lower Ki67 at all preprogression time‐points predicted for longer DoR. ER and PR significantly decreased in all tumours by 6 months (*p* < 0.001), with some declines in ER (serine 118) phosphorylation and Bcl‐2 (*p* = 0.007). There were modest HER2 increases (*p* = 0.034, 29% tumours) and loss of any detectable EGFR phosphorylation (*p* = 0.024, 50% tumours) and MAP kinase (ERK1/2) phosphorylation (*p* = 0.019, 65% tumours) by 6 months. While ER remained low, there was some recovery of Ki67, Bcl‐2 and (weakly) EGFR/MAPK activity in 45–67% patients at progression. Fulvestrant's anti‐proliferative impact is related to DoR, but while commonly downregulating ER and indicators of its signaling and depleting EGFR/MAPK signaling in some patients, additional elements must determine response duration. Residual ER at fulvestrant relapse explains reported sensitivity to further endocrine therapies. Occasional modest treatment‐induced HER2 and weakly detectable EGFR/HER2/MAPK signaling at relapse suggests targeting of such activity might have value alongside fulvestrant in some patients. However, unknown pathways must drive relapse in most. Ki67 has biomarker potential to predict fulvestrant outcome and as a quantitative measure of response.

## Abstract 2

**What's New?**

The steroidal drug fulvestrant is a powerful antiestrogen, blocking the estrogen receptor‐alpha to a greater extent than tamoxifen. However, the development of drug resistance is a considerable problem with fulvestrant. For the first time, the authors of the present study examined the biological effects of fulvestrant therapy using sequential breast tumor biopsies from untreated, treated, and relapsed patients. While residual estrogen receptor activity and tumor cell proliferation were detected at relapse, no meaningful increases were found in EGFR/HER2/MAPK activity. Ki67 expression was associated with duration of response, indicating promise as a predictive biomarker for fulvestrant outcome.

**Abbreviations**
ABC: advanced breast cancer
AF‐1: activation function domain‐1
AI: aromatase inhibitor
AKT: protein kinase B
Bcl‐2: apoptosis regulator B‐Cell CLL/Lymphoma 2
CB: clinical benefit
CI: confidence intervals
CONR/LP: continuing responders/late progressors (CB tumours with responses exceeding median DoCB)
DoCB: duration of response in patients with clinical benefit
DoR: duration of response in All‐patients
EGFR: epidermal growth factor receptor
EP: early progressors (CB tumours progressing prior to median DoCB)
ER: estrogen receptor‐alpha
ERK1/2: extracellular signal regulated kinases 1 and 2
GF: growth factor
HER2: receptor tyrosine‐protein kinase erbB‐2
HER3: receptor tyrosine‐protein kinase erbB‐3
HER4: receptor tyrosine‐protein kinase erbB‐4
IHC: immunohistochemistry
Ki67: proliferation‐related KI‐67 antigen
LAPC: locally advanced breast cancer
MAPK: mitogen‐activated protein kinase
mTOR: mammalian target of rapamycin
PD: progressive disease
PI3K: phosphatidylinositol 3‐kinase
PR: progesterone receptor
pS2: trefoil factor 1
Src: tyrosine‐protein kinase Src
TIMP‐1: tissue inhibitor of metalloproteinases 1

Fulvestrant (Faslodex™) is a pure anti‐estrogen with no known agonistic activity, contrasting tamoxifen. The steroidal agent fulvestrant prevents estradiol binding to estrogen receptor‐alpha (ER) to a stronger extent than tamoxifen. It also has a distinct mode of action that causes severe receptor conformational changes, promoting receptor degradation and downregulation of ER protein level and depletion of ER transcriptional activation.^1^ Clinically, therefore, fulvestrant retains activity in postmenopausal tamoxifen or nonsteroidal aromatase inhibitor (AI) resistant estrogen receptor positive (ER+) breast cancers.^2^, ^3^, ^4^, ^5^ Fulvestrant, at 250 mg, had similar time to progression, survival and response rate to use of tamoxifen/AIs in Phase III trials.^2^, ^3^, ^4^, ^6^ Following observations of dose‐dependent decline in ER after short‐term fulvestrant treatment of clinical breast cancer,^7^ additional studies including the CONFIRM trial^5^ provided evidence of further benefit with fulvestrant at 500 mg in ER+ disease following prior endocrine failure. Clinical significance of fulvestrant is set to increase further since this anti‐hormone also has potential in neoadjuvant and first‐line advanced ER+ postmenopausal disease settings, evidenced by trials including NEWEST^8^ and FIRST,^9^, ^10^ respectively. Further studies are also exploring fulvestrant alongside AIs, exemplified by the FACT^11^ and SWOG trials.^12^

Despite its increasing clinical value, *de novo* and acquired resistance remains a significant problem with fulvestrant. This disease state is largely unexplored in the clinical setting. The erbB receptor family members EGFR and HER2, as well as mitogen‐activated protein kinase signaling activity (MAPK), can be elevated and growth contributory to acquired fulvestrant resistance *in vitro,* 13, 14 although this is not a unifying feature of all fulvestrant resistant models.^15^ These can also be reliant on further erbB receptors,^16^ Src kinase and PI3K/AKT/mTOR signaling.^17^, ^18^ Moreover, growth factor (GF) signaling pathways have been heavily implicated in tamoxifen and estrogen deprivation resistance models, where they cross‐talk with ER. For example, EGFR/HER2 and MAPK signaling onto ER, *via* activation function domain 1 (AF‐1) residue phosphorylation (*e.g.,* serine 118), can permit either agonistic behaviour of tamoxifen or hypersensitivity to residual estrogens *in vitro*.^19^, ^20^, ^21^, ^22^ Theoretically, depletion of ER and thereby cross‐talk's critical “hub” should occur with fulvestrant, so that the development of resistance would potentially be delayed and possibly also ER‐independent. Fulvestrant is certainly able to promote ER degradation, decrease ER‐regulated proteins (*e.g.,* progesterone receptor [PR], pS2, cell survival protein Bcl‐2) and proliferation, and delay resistance in ER+ models.^23^ In short‐term studies (<16weeks) fulvestrant also decreased ER, PR and pS2, proliferation and (modestly) increased apoptosis in patient samples.^7^, ^8^, ^24^, ^25^ Nevertheless, some fulvestrant resistant patients retain sensitivity to further endocrine challenge.

Clearly, if we are to better understand response and acquired fulvestrant resistance in patients, it remains important to profile ER expression/function and GF signaling pathways during long‐term fulvestrant treatment. Indeed, it is our hypothesis that knowledge of such profiles through to fulvestrant resistance should aid interpretation of various breast cancer trials examining fulvestrant with anti‐GFs, could provide rationale for development of new strategies to delay or treat this resistant state, and may identify predictive biomarkers to maximize benefit from fulvestrant. Sequential breast cancer biopsies taken from locally advanced disease with or without metastases, prior to, during first‐line fulvestrant treatment and at subsequent relapse provide an important resource to help achieve this goal.

Here, for the first time, we profile the impact of initial (6 weeks) and prolonged (6 months and beyond) fulvestrant treatment (250 mg/month) on key elements of ER and GF signaling cross‐talk and proliferation in clinical ER+ samples. The immunohistochemical (IHC) methodology employed provides an immediate indication of potential value and feasibility of the various biomarker assays to predict fulvestrant clinical outcome.

## Material and Methods

Sequential core biopsies were obtained from 32 ER+ locally advanced or systemically advanced breast cancer patients treated with first‐line fulvestrant (250 mg/month). Thirty patients were from Faslodex™ 003 (an open label first‐line study to enable exploratory biological investigation; Nottingham Research Ethics Committee EC00/191). Two patients (with all sequential biopsies on unblinding) were from Faslodex™ 0025 (a randomized, double blind Phase III trial comparing 250 mg fulvestrant with 20 mg tamoxifen as first‐line therapy; EC98/239). Table 1 details criteria defining quality and duration of fulvestrant clinical response. Supporting Information Table S1a summarizes the patient series including baseline disease characteristics and clinical response (provided on a per case basis in Supporting Information Table S1b). The patient series showed good fulvestrant responses with a median duration of response (DoR) of 25.8 (1.8–60.7) months. Twenty‐six patients (81.25%) had clinical benefit (CB), with a median DoR in CB patients (DoCB) of 29.3 (10.9–60.7) months. Responses to any other treatments following fulvestrant progression were not a component of these response data. The median duration of overall survival (reflecting impact of first‐line fulvestrant and any subsequent disease management) was 35.5 (2.1–71.9) months, with 11 breast cancer‐specific deaths at analysis.

**Table 1:** Criteria defining quality and duration of response to fulvestrant

**QUALITY OF CLINICAL RESPONSE**
Patients were assessed clinically every 6 weeks for the first 6 months using bi‐dimensional calliper measurements of their tumour and then at 12 weekly intervals (as per UICC criteria): CR = Complete response to fulvestrant PR = Partial response to fulvestrant SD = Stable disease on fulvestrant CB = clinical benefit on fulvestrant, *i.e*., Complete or Partial response or Stable disease for >= 6 months PD = progressive disease, *i.e.,* progression of disease on fulvestrant within 6 months
**CLINICAL RESPONSE DURATION**
Median DoR = median duration of response between fulvestrant treatment commencement and disease progression on this agent for All‐patients Median DoCB= median duration of response to fulvestrant in patients with CB CB patients were also subdivided into EP (early progressors, *i.e*., CB tumours progressing prior to median DoCB on fulvestrant) or CONR/LP sub‐sets (continuing responders/late progressors, *i.e*., CB tumours with fulvestrant responses exceeding median DoCB, including those progressing on fulvestrant after this time). Two CB patients with follow‐up <median DoCB were excluded from EP versus CONR/LP analyses.

Core biopsies were taken from each tumour using a 14‐gauge needle (AA, JFR, KLC, EG) before commencing fulvestrant (T1), at 6 weeks (T2) and 6 months (T3) on treatment, and at disease progression (T4) for routine formalin‐fixation and paraffin‐embedding. Adequate cellularity (>100 tumour cells) was first verified in each biopsy (*n* = 31, 28, 25, 15 samples at T1‐T4, respectively) and cellularity was recorded. IHC was then performed on 3 μm sections for proliferation (Ki67) and biomarkers indicative of ER function [ER, PR, Bcl‐2, pER (serine 118 phosphorylated ER)], GF signaling [EGFR and HER2 expression or activity (Tyr845 and Tyr1248 phosphorylated pEGFR and pHER2, respectively)] or MAPK activity [Thr202/Tyr204 pMAPK, detecting phosphorylated MAPK1/3 (ERK1/2)]. HercepTest^TM^ and an additional assay with increased sensitivity for cytoplasmic staining monitored HER2 expression. All sequential samples for a patient were immunostained simultaneously. Semiquantitative assessment was performed by consensus of two observers blinded to patient details (percentage positivity for Ki67 and Bcl‐2; 0–3 HercepTest^TM^ scoring; H Score on a 0–300 scale for all other markers). Staining was nuclear for ER, PR, pER, pMAPK and Ki67, cytoplasmic for Bcl‐2, or plasma membrane (EGFRm, HER2m) and cytoplasmic (EGFRc, HER2c). Any samples with insufficient cellularity or nonspecific staining after each assay were excluded. The IHC staining and assessment (using standardized and internally validated protocols) is further detailed in Supplementary Assay Information.

Focussing on the All‐patient (*n* = 32) and clinical benefit (CB, *n* = 26) groups, early progressors (EP, *n* = 11) and continuing responders/late progressors (CONR/LP, *n* = 13) as defined in Table 1, biomarker changes were analyzed between matched T1, T2, T3 and T4 biopsies in each patient using Friedman's ANOVA and Wilcoxon Paired Signed‐Rank test (significance *p* <= 0.05). At each biopsy time point, Kaplan–Meier analysis (Log rank test) determined biomarker relationship to DoR on fulvestrant using the respective median staining cut‐point (Supporting Information Table S2), with disease progression on fulvestrant as the event. Staining relationship to DoCB was determined by Mann Whitney analysis in EP versus CONR/LP. Patient numbers were insufficient for analysis (*i*) within *de novo* fulvestrant resistant disease (PD, *n* = 6) or within specific CB patient subgroups and (*ii*) with respect to DoR according to T4 biomarker expression.

## Results

**Figure 1:**
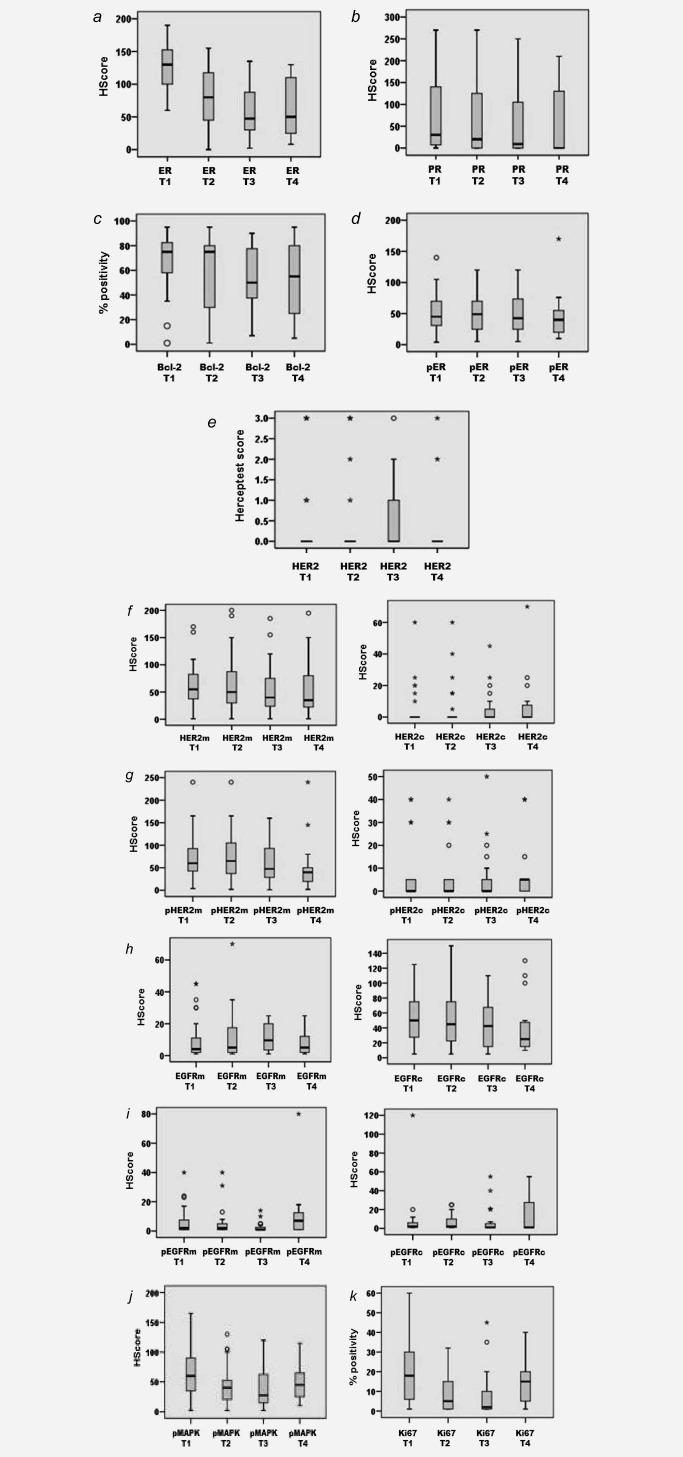
Box and whisker plots displaying marker profile (unmatched data) across T1 (pre‐treatment) and T2, T3 and T4 (6 week, 6 month and at progression on fulvestrant, respectively) for the All‐patient cohort. (*a*) ER H Score; (*b*) PR H Score; (*c*) % Bcl‐2; (*d*) pER (serine 118 phosphorylation) H Score; (*e*) HercepTest™ score; (*f*) HER2m (membrane) and HER2c (cytoplasmic) H Scores; (*g*) pHER2m (membrane) and pHER2c (cytoplasmic) H Scores; (*h*) EGFRm (membrane) and EGFRc (cytoplasmic) H Scores; (*i*) pEGFRm (membrane) and pEGFRc (cytoplasmic) H Scores; (*j*) pMAPK H Score and (*k*) % Ki67 staining (o = outliers and *= extreme outliers with values more than 3× the box height).

**Figure 2:**
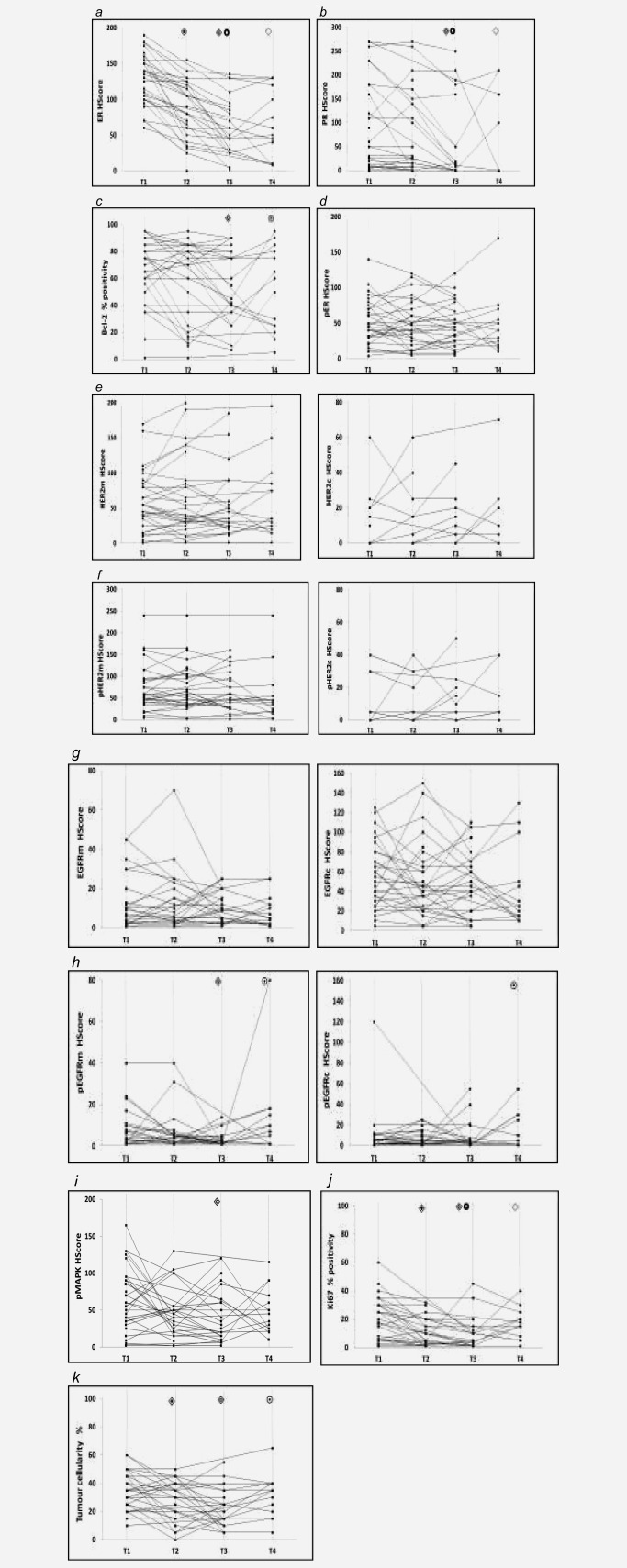
Line plots of individual changes in markers (matched data) across T1 (pretreatment) and T2, T3 and T4 (6 week, 6 month and at progression on fulvestrant respectively) for each patient. Wilcoxon Paired Signed‐Rank test compared staining between time‐points using paired sample data from each patient. Significance between T1‐T2, T1‐T3, T2‐T3, T1‐T4 or T3‐T4 is indicated by 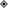, 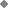, 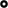, 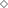 and 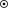, respectively. To aid visualisation of profile over the multiple time‐points in these plots, marker data have been connected in the same patient if any sample was unavailable. (*a*) ER H Score (T1‐T2, T1‐T3, T2‐T3 falls *p* < 0.001; T1‐T4 fall *p* = 0.001); (*b*) PR H Score (T1‐T3 *p* < 0.001, T2‐T3 falls *p* = 0.006; T1‐T4 fall *p* = 0.012); (*c*) Bcl‐2% positivity (T1‐T3 fall *p* = 0.007; T3‐T4 increase *p* = 0.066); (*d*) pER (serine 118 phosphorylation) H Score; (*e*) HER2m (membrane) and HER2c (cytoplasmic) H Scores; (*f*) pHER2m (membrane) and pHER2c (cytoplasmic) H Scores (NB. no measurable staining was detected in any sample from 20 patients for HER2c and 16 patients for pHER2c); (*g*) EGFRm (membrane) and EGFRc (cytoplasmic) H Scores; (*h*) pEGFRm (membrane; T1‐T3 fall *p* = 0.024; T3‐T4 increase *p* = 0.012) and pEGFRc H Scores (cytoplasmic; T3‐T4 increase *p* = 0.041); (*i*) pMAPK H Score (T1‐T3 fall *p* = 0.019); (*j*) Ki67% positivity (T1‐T2 *p* = 0.001, T1‐T3 *p* = 0.012, T2‐T3 falls *p* = 0.048; T1‐T4 fall *p* = 0.028); (*k*) Tumour cellularity % (T1‐T2 fall *p* = 0.019; T1‐T3 fall *p* = 0.003; T3‐T4 increase *p* = 0.065).

Figure 1 (with Supporting Information Table S2) shows the median staining obtained for the IHC biomarkers at each treatment time‐point in the All‐patient group. Staining data for individual patients are shown in Figure 2. Supporting Information Tables S3 and S4 provide median staining data for the CB (clinical benefit) and the EP (early progressors) and CONR/LP (continuing responders/late progressors) cohorts, respectively. Table 2 summarizes the key statistical findings for the biomarkers in these various patient cohorts during treatment.

**Table 2:** Summary of significant results from matched statistical analysis of biomarkers in the fulvestrant treated series for the All‐patients, CB (clinical benefit), EP (early progressors) and CONR/LP (continuing responders/late progressors) cohorts

	**All‐patients**	**CB**	**EP and CONR/LP**
**ER HScore**	T1‐T2‐T3: ER fall, *p* < 0.001 T1‐T2 or T1‐T3: ER fall, *p* < 0.001 T2‐T3: ER fall, *p* < 0.001 T1‐T4: ER fall, *p* = 0.001	T1‐T2‐T3: ER fall, *p* < 0.001 T1‐T2 or T1‐T3: ER fall, *p* < 0.001 T2‐T3: ER fall, *p* < 0.001 T1‐T4: ER fall, *p* = 0.004	**EP →** ER fall, T1‐T2: *p* = 0.018 T1‐T3: *p* = 0.003 T2‐T3: *p* = 0.028 **CONR/LP →** ER fall, T1‐T2: *p* = 0.003 T1‐T3: *p* = 0.005 T2‐T3: *p* = 0.008
**PR HScore**	T1‐T2‐T3: PR fall, *p* < 0.001 T1‐T2: PR unchanged in many patients T1‐T3: PR fall, *p* < 0.001 T2‐T3: PR fall, *p* = 0.006 T1‐T4: PR fall, *p* = 0.012	T1‐T2‐T3: PR fall, *p* < 0.001 T1‐T2: PR unchanged in many patients T1‐T3: PR fall, *p* = 0.008 T2‐T3: PR fall, *p* = 0.042 T1‐T4: PR fall, *p* = 0.018	**EP →** PR fall, T1‐T3: *p* = 0.008 T2‐T3: *p* = 0.042 **CONR/LP →** PR fall, T1‐T3: *p* = 0.093 T2‐T3: *p* = 0.063
**Bcl‐2%**	T1‐T2: Bcl‐2 unchanged in many patients T1‐T3: Bcl‐2 fall, *p* = 0.007 T3‐T4: Bcl‐2 rise in 64% patients, *p* = 0.066	T1‐T2: Bcl‐2 unchanged in many patients T1‐T3: Bcl‐2 fall, *p* = 0.012 T3‐T4: Bcl‐2 rise in 70% patients, *p* = 0.04	**EP →** Bcl‐2 fall, T1‐T2; *p* = 0.042 T1‐T3: *p* = 0.028 **CONR/LP →** T1‐T2: unchanged T1‐T3: *p* = 0.138
**pER HScore**	54% patients show decrease by T3, but no dominant change in pER during treatment	Some decreases in patients by T3, but no dominant change in pER during treatment	Some decreases in patients by T3, but no dominant change in pER during treatment
**HER2 expression**: HercepTest^TM^ or HER2m and HER2c H Score **HER2 activity**: pHER2m and HER2c H Score	HercepTest™: T1‐T2‐T3: HER2 rise, *p*< 0.001 T1‐T3: HER2 rise, *p* = 0.034	HercepTest™: T1‐T3: HER2 rise, *p* = 0.034 HER2c assay: T2‐T3: HER2c rise, *p* = 0.041	pHER2m assay: **EP →** pHER2m rise, T2‐T3: *p* = 0.078
**EGFR expression**: **EGFR activity**: pEGFRm andpEGFRc H Score	EGFR: no dominant change T1‐T3: pEGFRm fall, *p* = 0.024 T3‐T4: pEGFRm rise in 67% patients, *p* = 0.012 T3‐T4: pEGFRc rise in 50% patients, *p* = 0.041	EGFR: no dominant change T1‐T3: pEGFRm fall, *p* = 0.028 T3‐T4: pEGFRm rise in 72% patients, *p* = 0.011 T3‐T4: pEGFRc rise in 55% patients, *p* = 0.041	EGFR: No dominant change Some falls but no dominant change in pEGFR
**pMAPK Hscore**	T1‐T3: pMAPK fall, *p* = 0.019	T1‐T3: pMAPK fall, *p* = 0.028	Some falls but no dominant change in pMAPK
**Ki67%**	T1‐T2‐T3: Ki67 fall, *p* < 0.001 T1‐T2: Ki67 fall, *p* = 0.001 T1‐T3: Ki67 fall, *p* = 0.012 T2‐T3: Ki67 fall, *p* = 0.048 T1‐T4: Ki67 fall, *p* = 0.028 T1‐T2‐T3‐T4: Ki67 rise in 45% patients, *p* = 0.077	T1‐T2‐T3: Ki67 fall, *p* < 0.001 T1‐T2: Ki67 fall, *p* = 0.001 T1‐T3: Ki67 fall, *p* = 0.012 T2‐T3: Ki67 fall, *p* = 0.048 T1‐T4: Ki67 fall, *p* = 0.074	**EP →** Ki67 fall T1‐T2‐T3: *p* = 0.032 T1‐T2: *p* = 0.027 T1‐T3: *p* = 0.033 T2‐T3: unchanged **CONR/LP →** Ki67 fall T1‐T2‐T3: *p* = 0.003 T1‐T2: *p* = 0.021 T1‐T3: *p* = 0.007 T2‐T3: *p* = 0.021
**Tumour** **cellularity %**	T1‐T2‐T3: cellularity fall, *p* = 0.007 T1‐T2: cellularity fall, *p* = 0.019 T1‐T3: cellularity fall, *p* = 0.003 T3‐T4: cellularity rise in 50% patients, *p* = 0.065	T1‐T2‐T3: cellularity fall, *p* = 0.007 T1‐T2: cellularity fall, *p* = 0.01 T1‐T3: cellularity fall, *p* = 0.004 T3‐T4: cellularity rise in 46% patients, *p* = 0.088	**EP →** cellularity fall T1‐T2‐T3: *p* = 0.018 T1‐T2: *p* = 0.045 T1‐T3: *p* = 0.028 **CONR/LP →** cellularity fall T1‐T2‐T3: *p* = 0.067 T1‐T2: *p* = 0.057 T1‐T3: *p* = 0.027

Statistical analyses were performed in SPSS^TM^ using Friedman's ANOVA for T1‐T2‐T3 and T1‐T2‐T3‐T4 and Wilcoxon Paired Signed‐Rank test for all paired analysis. T1= pre‐treatment; T2= 6 week fulvestrant treatment; T3= 6 month fulvestrant treatment; T4= fulvestrant progression; c= cytoplasmic; m= membrane; CB= clinical benefit patient group; EP= early progressor patient group; CONR/LP= continuing responder/late progressor patient group.

### ER expression, activity and ER‐regulated proteins PR and Bcl‐2

ER declined significantly versus T1 at all fulvestrant treatment time‐points and in all cohorts reaching its lowest levels by T3 which were then generally maintained at T4 (Figs. 1 *a* and 2*a*; Table 2; Supporting Information Tables S2–S4). No patient had lost all ER at relapse even in the longest T1‐T4 interval of 60.7 months. An example of sequential biopsy ER immunostaining is provided in Supporting Information Fig. S1*a*.

PR was detectable in most patients at T1. Despite small numbers of progressive disease patients, it was noted that PR was significantly lower than in CB patients at T1 (*p* = 0.012), with three of the 4 PR‐negative tumours being progressive disease. Although falls were generally modest at T2, by T3 PR had declined in all of the patient cohorts versus T1 (Figs. 1 *b* and 2*b*; Table 2; Supporting Information Tables S2–S4). Supporting Information Fig. S1*b* shows an example of sequential biopsy PR immunostaining. Interestingly, PR fall at T2 was more substantial and by T3 reached significance in EP patients, contrasting a more modest decline in the longer‐responding CONR/LP patients (Table 2; Supporting Information Table S4). PR remained significantly lower at T4 than T1 in the All‐patient cohort (Figs. 1 *b* and 2*b*; Supporting Information Table S2) and CB cohort following matched analysis **(**Table 2), with complete PR loss in one third of tumours (as predominantly seen in Supporting Information Fig. S1*b*). However, three tumours maintained substantial PR (H‐Score >= 100) at T4 exceeding T3 (Fig. 2 *b*).

Bcl‐2 was detected in all T1 samples irrespective of response status. Bcl‐2 fell significantly in 58% patients by T3 (Figs. 1 *c*, 2*c*; Table 2; Supporting Information Tables S2, S3 and Supporting Information Fig. S1*c*). Significant Bcl‐2 decreases were apparent in early progressors at both T2 and T3, but there was no T2 fall in CONR/LP patients and a marginal decline by T3 (Table 2; Supporting Information Table S4). Some T3‐T4 Bcl‐2 recovery occurred in approximately 65% patients (Fig. 2 *c*), reaching significance in about 70% CB so there was no significant staining difference between T1 and T4 (Table 2; Supporting Information Table S3). Supporting Information Figure S1*c* shows such Bcl‐2 staining.

ER activity (phosphorylated serine 118), again detected in all T1 samples irrespective of response status, decreased very modestly in 54% patients by T3 on matched analysis (Fig. 2 *d*; Table 2). Supporting Information Figure S1*d* provides an example of such sequential biopsy pER staining. No patient was pER negative at T4 and there was no consistent pattern of pER change at this time‐point (Figs. 1 *d*, 2*d*; Supporting Information Tables S2 and S3). Spearman's analysis showed that pER weakly correlated with ER expression at all time‐points (T1 *p* = 0.018, *r* = 0.42; T2 *p* = 0.054, *r* = 0.38; T3 *p* = 0.025, *r* = 0.46; T4 *p* = 0.003, *r* = 0.74) and with Bcl‐2 at T1 (*p* = 0.005, *r* = 0.5) and T3 (*p* = 0.008, *r* = 0.53).

**Figure 3:**
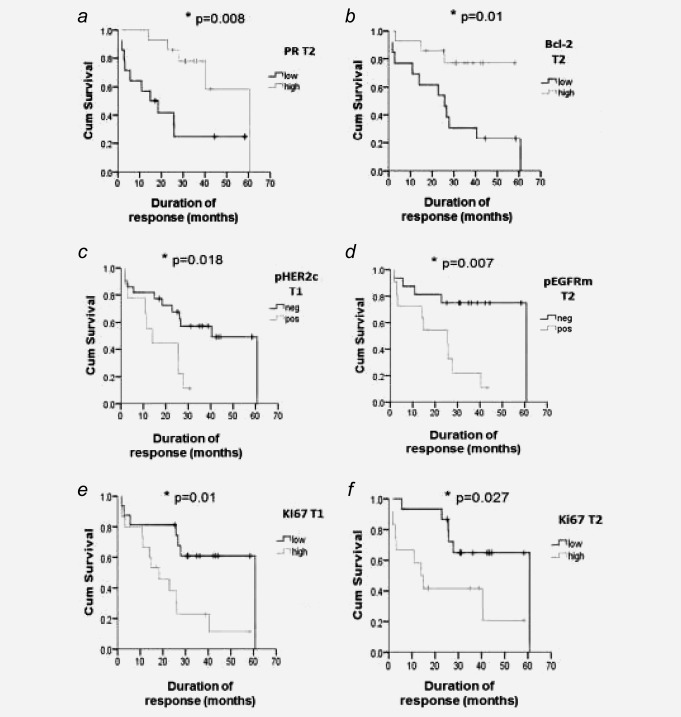
Kaplan–Meier association (using the median staining level from Supporting Information Table S2 at each time point as a cut‐point) between biomarkers and duration of response (DoR) to fulvestrant (*p < 0.05 using Log Rank test). Responses to any other treatments following fulvestrant progression were not a component of these response data. (*a*) PR H Score at T2; *p* = 0.008 for higher PR [mean DoR= 48.2 months (95% CI=36.9–59.5; *n* = 14)] *vs*. lower PR [mean DoR= 23.4 months (95% CI= 11.6–35.1; *n* = 14)]; (*b*) Bcl‐2% at T2; *p* = 0.01 for higher Bcl‐2% [mean DoR= 48.4 months (95% CI=38.5–58.4; *n* = 13)] *vs*. lower Bcl‐2% [mean DoR= 27.5 months (95% CI= 15.3–39.6; *n* = 14)]; (*c*) pHER2c (cytoplasmic) H Score at T1; *p* = 0.018 for any detectable pHER2c [mean DoR= 16.8 months (95% CI= 10.1–23.5); *n* = 9] versus no pHER2c [mean DoR= 39.1 months (95% CI= 28.5–49.7; *n* = 22)]; (*d*) pEGFRm (membrane) H Score at T2; *p* = 0.007 for any detectable pEGFRm [mean DoR= 21.1 months (95% CI= 12.5–29.7; *n* = 11)] *vs*. no pEGFRm [mean DoR= 48.1 months (95% CI= 36.0–60.3; *n* = 16)]; (*e*) Ki67% at T1; *p* = 0.01 for higher Ki67 [mean DoR= 22.2 months (95% CI= 13.2–31.3; *n* = 15)] *vs*. lower Ki67 [mean DoR= 43.1 months (95% CI= 30.6–55.5; *n* = 16)]; (*f*) Ki67% at T2; *p* = 0.027 for higher Ki67 [mean DoR= 24.7 months (95% CI= 11.6–37.9; *n* = 12)] *vs*. lower Ki67 [mean DoR= 47.1 months (95% CI= 36.2–58.0; *n* = 15)].

Kaplan–Meier analysis revealed no significant relationship between ER expression or activity and DoR on fulvestrant at any time‐point or at T1 or T3 for PR or Bcl‐2 (data not shown). However, DoR was significantly prolonged where PR or Bcl‐2 staining at T2 exceeded the median cut‐point [Figs. 3 *a* (*p* = 0.008) and 3*b* (*p* = 0.01), respectively], while Bcl‐2 level in CONR/LP patients also significantly exceeded early progressors at this time‐point (*p* = 0.01; Supporting Information Table S4).

### HER2, EGFR and their activated forms (pHER2, pEGFR)

Generally, very modest HER2 membrane (HER2m) expression and activity (pHER2m) were detectable in this series, with little or no HER2 cytoplasmic (HER2c, pHER2c) staining (Figs. 1 *e*‐*g*; Figs. 2*e* and 2*f*; Supporting Information Tables S2–S4). Staining with HercepTest^TM^ was seen at T1 in 17% patients (5 of the 29 patients with adequate HercepTest^TM^ data), including three 3+ samples comprising one CB and two PD. T1 samples with HercepTest^TM^ score >0 also showed some positivity for HER2m/pHER2m. There were few significant changes in HER2 expression or activity with fulvestrant in this patient series (Figs. 1 *e*‐*g*; Figs. 2*e* and 2*f*; Supporting Information Tables S2–S4; Supporting Information Figs. S1*e* and S1*g*). HercepTest^TM^ staining increased in 29% patients by T3 (Fig. 1 *e*). These increases reached significance by matched analysis in the whole series and in CB patients (Table 2) but were modest (HercepTest^TM^ score 0‐to‐1: *n* = 2; 0/1‐to‐2: *n* = 3) with no 3+ gains. An additional HER2 expression assay also detected T2‐T3 HER2c increases in approximately 25% paired samples from the whole series (Figs. 1 *f* and 2e), although many remained HER2c negative. This increase reached significance in CB (Table 2), where there were additionally occasional modest T3 increases in HER2m and pHER2 on matched analysis in approximately 40% of patients (Figs. 2 *e* and 2 *f*; Supporting Information Figs. S1*f* and S1*h*). There was a trend for a T2‐T3 pHER2m increase in 60% EP samples (Table 2). HER2m expression (*p* = 0.028, *r* = 0.45) and activity at T3 (*p* = 0.016, *r* = 0.49) directly associated with pER by Spearman's analysis. Although occasional samples showed changes (including slight HER2c and pHER2c increases and one patient with a 0‐to‐2 HercepTest^TM^ score increase), there was no dominant pattern in HER2 expression/activity at relapse with generally modest levels in T4 samples (Figs. 1 *f* and 1 *g*; Figs. 2*e* and 2*f*; Supporting Information Tables S2 and S3; Supporting Information Figs. S1*e* and S1*g*).

This series also had low EGFR membrane (EGFRm) and cytoplasmic (EGFRc) staining, excepting four patients at T1 (H Score>=100) of which three were CB. Some EGFR changes were seen in samples during treatment but membrane H Scores remained very low (median <=10) along with modest cytoplasmic signals (Figs. 1 *h* and 2*g*; Supporting Information Fig. S1*i*; Supporting Information Tables S2–S4). There was no dominant pattern of EGFR expression change on matched analysis at any time‐point (Fig. 2 *g*; Table 2). Similarly, pEGFR levels were generally very low (median <=10; Fig. 1*i*; Supporting Information Tables S2–S4). There was significant loss of detectable pEGFRm staining in approximately 50% patients by T3 including in CB (Figs. 1 *i* 2*h*; Table 2; Supporting Information Fig. S1*j*). While still generally at extremely low levels at T4 (median H Score <=10), small significant increases were subsequently detected for pEGFRc or pEGFRm in 50–67% T4 samples including 72% of CB (Figs. 1 *i* and 2*h*; Table 2; Supporting Information Tables S2 and S3; Supporting Information Fig. S1*j*). Tumours with weakly detectable T3‐T4 increases in pEGFRm also showed modest HER2m increases (*p* = 0.027) and Spearman's analysis revealed direct associations at T4 between EGFR activity and pER (*p* = 0.006), Bcl‐2 (*p* = 0.055) and Ki67 (*p* = 0.047).

Kaplan–Meier analysis was not meaningful for total EGFR or HER2 expression since this ER+ series contained very few highly HER2 or EGFR expressing tumours. Patients with any pHER2c positivity at T1 had a shortened DoR (*p* = 0.018; Fig. 3 *c*) but no relationship was seen for pHER2m. Although levels were extremely low, a shortened DoR was also observed for patients with any detectable pEGFRm at T2 (*p* = 0.007; Fig. 3 *d*). pHER2c (*p* = 0.051) and pEGFRm (*p* = 0.037) were also weakly increased in EP versus CONR/LP patient samples at these respective time‐points.

### MAPK (ERK1/2) activity

Nuclear MAPK activity (pMAPK), detected at moderate levels in T1 samples irrespective of response status, significantly declined in approximately 65% of All‐patient and CB samples at T3 (Figs. 1 *j*, 2*i*; Table 2; Supporting Information Tables S2 and S3; Supporting Information Fig. S1*k*). Some falls were also apparent in EP and CONR/LP patients (Supporting Information Table S4). Such tumours with a pMAPK decrease by 6 months (*n* = 16) also showed significant decline in pEGFRm (*p* = 0.013), Bcl‐2 (*p* = 0.031) and Ki67 (*p* = 0.003). Subsequent non‐significant nuclear pMAPK increases occurred in approximately 50% T4 samples versus T3 (Figs. 1 *j*, 2*i*; Supporting Information Tables S2 and S3; Supporting Information Fig. S1*k*). Kaplan–Meier analysis revealed no relationship between pMAPK and DoR (data not shown).

### Proliferative activity (Ki67)

Ki67 staining was modest for most samples, with significant decreases across T1‐T2‐T3 during fulvestrant treatment in all groups (Figs. 1 *k* and 2*j*; Table 2; Supporting Information Tables S2–S4; Supporting Information Fig. S1*l*). T2 and T3 Ki67 positivity was significantly lower than T1 in up to 79% patients for all groups (Fig. 2 *j*; Table 2). Tumour cellularity also decreased during fulvestrant response with T2 and T3 level significantly lower than T1 on matched analysis (Table 2; Fig. 2*k*; Supporting Information Tables S2–S4). There was a further very small but significant T2‐T3 Ki67 fall in approximately 60% of the All‐patient and CB cohorts (Figs. 1 *k* and 2*j*; Table 2). Interestingly, this continued T2‐T3 decline was only seen in the longer‐responding CONR/LP cohort and not in early progressors, where by T3 there was an indication of some modest recovery in proliferation (Table 2; Supporting Information Table S4). Although T4 Ki67 remained a little lower than T1 for approximately 70% patients (Figs. 1 *k*, 2*j*; Table 2) there was partial recovery in Ki67 in some T4 samples (Table 2: T1‐T2‐T3‐T4 *p* = 0.077; Fig. 2*j*; Supporting Information Tables S2 and S3; Supporting Information Fig. S1*l*) with T3‐T4 increases in 5/11 (about 45%) CB patients. Tumour cellularity also recovered in approximately 50% patients at T4 (Fig. 2 *k*; Table 2; Supporting Information Tables S2 and S3).

Kaplan–Meier analysis demonstrated a significantly shortened DoR at T1 (*p* = 0.01) for patients with Ki67 above the median cut‐point (>18% staining; Fig. 3 *e*). Concordantly, Ki67 was at a significantly higher level in early progressors versus the longer‐responding CONR/LP patient cohort at T1 (*p* = 0.037; Supporting Information Table S4). Multivariate analysis using Cox's proportional hazards model [considering univariate‐significant covariates baseline disease site and grade (Supporting Information Table S1), T1 pHER2c and T1 Ki67 (Figs. 3 *c* and 3 *e*)] showed T1 Ki67 was an independent predictor of fulvestrant DoR (*p* = 0.012), with high staining patients having a hazard 6.6‐fold that of low staining patients (Supporting Information Table S5). While levels were very low at T2 and T3 in CB patients, retention of any Ki67 at T2 was also adversely associated with DoR (*p* = 0.027; Fig. 1 *f*). With a trend at T2 (*p* = 0.071), early progressors had a significantly higher Ki67 level at T3 versus the longer‐responding CONR/LP cohort (*p* = 0.043; Supporting Information Table S4).

## Discussion

This is the first clinical investigation of the biological impact of short (6 weeks), medium (6 months) and long‐term (>2 years) fulvestrant (250 mg/month) through to acquired resistance using sequential biopsies. In this ER+ breast cancer series, there was superior benefit for fulvestrant, with 81% CB and a median DoR of 25.8 (1.77–60.73) months, compared with previous reports of up to 60% CB and 4‐18 months response.^3^, ^4^, ^6^, ^26^, ^27^ Our series had substantial ER‐regulated proteins, modest EGFR/HER2 signaling and elevated Ki67 expression, a profile similarly equated to better outcome for other anti‐hormones.

The ER downregulation we observed with fulvestrant at 6 weeks is consistent with previous short‐term (<3 weeks) pre‐surgical primary breast cancer studies for short‐acting (6 or 18 mg/daily subcutaneously^28^) and long‐acting formulations (50–250 mg/month intramuscularly^7^). ER decline is also seen in the neoadjuvant (4 and 16 weeks treatment) setting.^8^ Such studies demonstrated that ER downregulation is fulvestrant dose‐dependent but we demonstrate here that treatment duration is a further influence since superior ER decline was achieved by 6 months (the timeframe for 250 mg fulvestrant to reach steady‐state^2^). Critically, we have found that ER level at all time‐points fails to relate to fulvestrant response. Indeed, by 6 weeks virtually every tumour had significant ER decline irrespective of patient response status or duration [occurring in CB (clinical benefit), PD (progressive disease), EP (early progressors) and CONR/LP patients (continuing responders/late progressors)]. The CONFIRM trial^5^ demonstrated a longer duration of CB while the NEWEST trial^8^ reported greater ER depletion for 500 mg versus 250 mg fulvestrant. However, our findings indicate that despite ER being the required target for fulvestrant and ER downregulation a hallmark of this agent's mechanism of action, parameters other than receptor level must determine extent of clinical fulvestrant response in ER+ tumours.

To determine whether ER activity was more informative, we monitored fulvestrant impact on ER phosphorylation and two ER‐regulated proteins. Although patient number precluded meaningful analysis of pER or Bcl‐2, PD patients were commonly PR negative at baseline suggesting classical estrogen/ER signaling is needed to achieve CB with fulvestrant. However, these PR findings remain clinically controversial.^29^, ^30^ Moreover, tissue inhibitor of metalloproteinases 1 (TIMP‐1) overexpression has been noted to promote PR loss and fulvestrant resistance *in vitro* potentially *via* modifying nonclassical ER activity.^31^ Fulvestrant promoted a time‐dependent fall in PR and Bcl‐2 (and modest pER decline in about 50% patients) in our series by 6 months. Previous shorter‐term studies using 250 mg fulvestrant have reported significant PR decline,^7^, ^32^ but our findings again equate better with the timeframe for 250 mg dose steady state^2^ and corroborate with NEWEST trial observations that 500 mg is required to significantly repress PR during short‐term treatment.^8^ We found that baseline ER activity, PR and Bcl‐2 did not relate to duration of CB, and in the longer‐responding (CONR/LP) patients PR and Bcl‐2 decline during treatment was at best small. This suggests that an extended DoR with fulvestrant does not equate with superior blockade of these particular ER‐regulated proteins.

We also examined whether fulvestrant influenced ER/GF pathway cross‐talk and if this determined response. Experimentally, endocrine agents can deplete ER‐regulated growth factor ligands for upstream receptors of MAPK,^22^ and after 6 months fulvestrant we noted that any membrane EGFR activity was lost and MAPK (ERK1/2) activity decreased. Such pMAPK depletion may contribute toward the small fall in phosphorylated serine 118 ER seen in some fulvestrant‐treated patients since pMAPK activates this AF‐1 residue.^22^ This pMAPK fall after 6 months fulvestrant was paralleled by Ki67 decline, so inhibition of ER/MAPK cross‐talk may contribute towards fulvestrant's anti‐proliferative effect, as reported in some ER+ models.^33^, ^34^ However, as T3 pMAPK and pEGFR decreases occurred in only about one‐half of CB patients, were extremely modest for pEGFR and were unrelated to outcome, further mechanisms must contribute to fulvestrant response in patients.

Importantly, low baseline Ki67 (<=median 18%) significantly associated with durable fulvestrant response in univariate and multivariate analysis. The subsequent fall in proliferation in many patients by 6 weeks was consistent with previous short‐term fulvestrant studies^7^, ^8^, ^28^ but we also determined that patients with the very lowest resultant proliferation (<=median 5%) had a longer DoR. In the IMPACT trial,^35^, ^36^ reduced proliferation at 2 weeks similarly predicted for extended disease‐free interval with tamoxifen or anastrozole presurgically. Proliferation suppression by fulvestrant was also reported in the neoadjuvant NEWEST trial^8^ with lowest Ki67 achieved using the clinically superior 500 mg dose.^5^ We also found that depletion of proliferation was apparent with longer‐term fulvestrant in many CB patients. By 6 months, there was a very small, continued Ki67 decline in the longer‐responding CONR/LP group contrasting partial recovery in early progressors. Thus, patients with the very lowest Ki67 after 6 months fulvestrant demonstrated superior response. Although further verification (including at 500 mg) is required, we propose that measuring this proliferation marker could have clinical predictive utility both to determine patients likely to substantially benefit from fulvestrant and as a quantitative measure of response.

We also report for the first time the tumour biomarker profile on acquisition of fulvestrant resistance in patients. Ki67 recovered in some clinical relapse samples compared with 6 month's treatment and this is likely to contribute towards tumour re‐growth (evidenced by increased cellularity). Frequent Bcl‐2 recovery suggests increased cell survival also plays a part. Low ER levels were retained at relapse, as seen in some acquired resistant models developed after 3–12 months fulvestrant treatment of ER+ cells.^14^ Several studies^37^, ^38^, ^39^ suggest that this residual ER may be functional in some patients. Cheung *et al*.^39^ reported CB following further endocrine treatment in 46% (13/28) and 12% (3/26) of patients who had initial CB or PD respectively on fulvestrant, including responses in seven patients that overlap with our series and retain ER at fulvestrant relapse. The persistent low levels of ER activity, PR and Bcl‐2 that we observed in fulvestrant relapse samples and the ER phosphorylation and Bcl‐2 detectable at pretreatment in PD patients provide further evidence for functional ER in some fulvestrant resistant tumours. This begs the question whether an increased dosage of fulvestrant might further deplete ER signaling and improve response, and provides rationale for development of more potent ER‐downregulators. In further support, superior ER depletion was seen with 500 mg fulvestrant both in NEWEST^8^ (*vs*. 250 mg examined up to 4 weeks) and Study 57.^40^ Treatment at 500 mg was also associated with improved DoCB and overall survival in CONFIRM^5^ and increased time to progression in the FIRST trial,^9^, ^10^ the latter also recently reporting better overall survival with fulvestrant versus anastrozole.^41^ Nevertheless, as Cheung *et al*.^39^ observed insensitivity to further endocrine agents in 54% of patients with initial CB on fulvestrant, a significant proportion of fulvestrant relapse patients may be

ER‐independent despite retention of ER. Moreover, some cell models reveal more prolonged (>2 years *in vitro*) fulvestrant can promote complete ER protein and mRNA loss.^42^ Theoretically, while ER positivity is a stable phenotype over the treatment window of the current study with 250 mg drug, further prolonging fulvestrant might ultimately promote an undesirable ER negative phenotype.

Increased EGFR/HER2 and MAPK signaling cross‐talks with ER AF‐1, promotes PR loss and drives tamoxifen or estrogen deprivation resistance *in vitro*.^43^ It is also detectable in some clinical samples on tamoxifen progression.^44^, ^45^ Here, very modest increases in EGFR activity occurred at relapse in approximately 65% fulvestrant treated patients versus 6 months, associating with HER2, pER, Bcl‐2 and proliferation. pMAPK also modestly recovered and PR was lost in about one‐third of relapse patients. Furthermore, although PD patients were few, two were HER2+ and one had high EGFR at pretreatment. Anti‐estrogen induced EGFR/HER2 signaling can begin to emerge during response in ER+ cells *in vitro*.^14^, ^33^, ^34^ Here, HercepTest^TM^ score modestly increased in about one‐quarter of fulvestrant‐treated patients by 6 months and HER2 activity also weakly increased (particularly in early progressors) and correlated with pER. Along with preclinical studies,^19^, ^33^, ^34^, ^46^ our findings suggest such GF signaling and its cross‐talk with residual ER might modestly contribute towards limiting response in a small number of fulvestrant‐treated patients. However, HER2 induction with fulvestrant was infrequent, relapse was not paralleled by substantially increased EGFR/HER2/MAPK activity, and PD did not obviously associate with such signaling since one ER+ HercepTest^TM^ positive patient and three patients with substantial baseline EGFR had clinical benefit on fulvestrant. These findings suggest lack of a central role for HER2/EGFR signaling in clinical fulvestrant resistance and explain recent trials showing no benefit of combining fulvestrant with lapatinib^47^ or gefitinib.^48^ Our findings also add to evidence that fulvestrant can be considered for HER2 or EGFR overexpressors.^29^, ^49^ For most patients, additional pathways clearly drive fulvestrant progression and pre‐clinical data are implicating potential players including erbB receptors,^13^, ^16^ PI3K/AKT/mTOR, Src kinase^17^, ^18^ and TIMP‐1.^31^ IHC in this sequential fulvestrant sample series is now continuing for further elements which should help interpret ongoing trials, including fulvestrant alongside PI3K or AKT inhibitors or with everolimus.^50^

## Supporting information

Supporting Information Figure s1

Supporting Information Tables s1ab‐s5

Supporting Information LAB methods
