# Supplementary material for: Biological effects of fulvestrant on estrogen receptor positive human breast cancer: short, medium and long‐term effects based on sequential biopsies
Source: Int J Cancer. 2015 Jul 30;138(1):146–59. doi: 10.1002/ijc.29682 (PMC4879515; doi:10.1002/ijc.29682)
Supplement: Supplementary file 2 — Supporting Information Tables s1ab‐s5 [file IJC-138-146-s002.rtf]

SUPPLEMENTARY TABLES: S1a, S1b, S2, S3, S4, S5.
Biological effects of fulvestrant on estrogen receptor positive human breast cancer: short, medium and long-term effects based on sequential biopsies. 
Agrawal A, Robertson JFR, Cheung KL, Gutteridge E, Ellis IO, Nicholson RI, Gee JMW.
Supplementary Table S1a: Summary of patient demographics, disease baseline characteristics, clinical response to fulvestrant and overall survival for the series.
PATIENTS AND DISEASE SITE		
Median age	74.2 (53.8-90.9) years	
Disease site
Local only (n=22); local with bone (n=8) with additional disease in lungs/pleura (n=3) or with liver (n=1); lung/pleura only (n=2) 	Locally advanced breast cancer; 
68.7 % (n=22); mean DoR= 42.3 months 
[95% CI= 32.5 - 52.1 months]
Advanced breast cancer; 
31.3% (n=10); mean DoR= 14.4 months 
[95% CI= 6.5 - 22.3 months];  p=0.001  	
BASELINE DIAGNOSTIC PATHOLOGY		
Tumour grade 	Grade 1= 23.3% (n=7); mean DoR= 42.4 months [95% CI= 23.6 - 61.2 months];

Grade 2= 66.7% (n=20); mean DoR= 33.5 months [95% CI= 24.6 – 42.4 months]; 
                    
Grade 3= 10.0% (n=3); mean DoR= 9.3 months [95% CI= 1.7 – 16.9 months];  p=0.01	
Histopathology (all invasive tumours)	Ductal no specific type NST= 71.9% (n=23)
Lobular = 21.9% (n=7)
Mixed ductal NST + lobular = 6.2% (n=2)	
CLINICAL RESPONSE QUALITY  TO FULVESTRANT		
CR= complete response
PR= partial response
SD= stable disease 
CB= clinical benefit (= CR+PR+SD)
PD= progressive disease	CR n=1,     3.12% 
PR n=8,     25% 
SD n=17,   53.12%
CB  n=26,  81.25%                                        PD  n=6,    18.75% 	
CLINICAL RESPONSE DURATION ON FULVESTRANT		
Median DoR  (duration of fulvestrant response for All-patients) 	25.8 (1.77 – 60.73) months	
Median DoCB (duration of fulvestrant response in CB patients)  	29.3 (10.9–60.73) months	
PATIENT OVERALL SURVIVAL		
Median overall duration of survival (from date of entry into the study to death or last follow-up)	35.5 (2.1 – 71.9) months	
Median duration of survival after fulvestrant withdrawal	10.9 (0 – 23.0) months	
Deaths	11 patients had died at time of analysis and 
all deaths were breast cancer-specific.	
Supplementary Table S1b: Disease baseline characteristics, clinical response to fulvestrant and overall survival by case number. 

LAPC = locally advanced breast cancer; ABC = advanced (metastatic) breast cancer; NST= ductal no specific type; CR= complete response; PR= partial response; SD= stable disease; PD= progressive disease; DoR= duration of response; DoCB= duration of CB (clinical benefit); EP= early progressor; CONR/LP= continuing responders or late progressors; S= CB patient with short follow-up time (<median DoCB).


Supplementary Table S2: Median level of markers at each biopsy time-point in All-patients.
Markers 
	Median T1 (Range); no.	Median T2 (Range); no.	Median T3 (Range); no.	Median T4 (Range); no.	
ER HScore	130 (60-190); 31	80 (0-155); 27	48 (2-135); 24	50 (8-130); 15	
PR HScore	30 (0-270); 31	20 (0-270); 28	9 (0-250); 20	0 (0-210); 12	
Bcl-2 %	75 (1-95); 31	75 (1-95); 27	50 (7-90); 24	55 (5-95); 14	
pER HScore        	45 (4-140); 31	49 (5-120); 26	43 (5-120); 24	40 (10-170); 14	
HER2c HScore	0 (0-60); 31	0 (0-60); 27	0 (0-45); 24	0 (0-70); 15	
HER2m HScore	55 (1-170); 31	50 (1-200); 27	40 (1-185); 24	35 (1-195); 15	
HercepTestTM 	0 (0-3); 29	0 (0-3); 25	0 (0-3); 21	0 (0-3); 11	
pHER2c HScore	0 (0-40); 31	0 (0-40); 27	0 (0-50); 24	5 (0-40); 15	
pHER2m HScore	60 (4-240); 31	65 (2-240); 27	48 (1-160); 24	40 (2-240); 15	
EGFRc HScore	50 (5-125); 31	45 (5-150); 27	43 (5-110); 24	25 (10-130); 15	
EGFRm HScore	4 (1-45); 31	5 (1-70); 27	10 (1-25); 24	5 (1-25); 15	
pEGFRc HScore 	2 (1-120); 31	2 (1-25); 27	1 (1-55); 24	1 (1-55); 15	
pEGFRm HScore	2 (1-40); 31	2 (1-40); 27	1 (1-14); 24	7 (1-80); 15	
pMAPK HScore	60 (2-165); 31	40 (2-130); 27	28 (2-120); 24	45 (10-115); 15	
Ki67 % 	18 (1-60); 31	5 (1-32); 27	2 (1-45); 24	15 (1-40); 15	
Tumour cellularity %	35 (10-60); 31	35 (0-50); 27	20 (5-55); 25	35 (5-65); 15	
T1= pre-treatment; T2= 6 week fulvestrant treatment; T3= 6 month fulvestrant treatment; 
T4= fulvestrant progression; no.=  patient numbers; c= cytoplasmic; m= membrane. 
Discrepancies between the patient numbers at each time-point and the All-patient cohort size (n=32) arose because of several factors: (i) in one patient although samples taken on treatment proved adequate, the T1 biopsy had inadequate cellularity (<100 tumour cells) for any biomarker analysis; 
(ii) since tumours receded during response, adequate T2/T3 samples could not be obtained for all patients; (iii) further discrepancies arose either as a consequence of inadequate cellularity or non-specific staining in occasional samples following performance of the particular immunohistochemical assay.


Supplementary Table S3: Median level of markers at each biopsy time-point in patients with clinical benefit (CB) on fulvestrant. 
Markers 
	Median T1 (Range); no.	Median T2 (Range); no.	Median T3 (Range); no.	Median T4 (Range); no.	
ER Hscore	135 (60-190); 25	93 (0-155); 22	50 (2-135); 23	50 (9-130); 11	
PR Hscore	50 (0-270); 25	25 (0-270); 23	10 (0-250); 19	50 (0-210); 8	
Bcl-2 %	75 (15-95); 25	75 (10-95); 23	55 (7-90); 23	70 (25-95); 10	
pER HScore        	50 (4-140); 25	44 (5-120); 22	45 (5-120); 23	45 (10-170); 10	
HER2c Hscore	0 (0-60); 25	0 (0-25); 22	0 (0-45); 23	0 (0-25); 11	
HER2m Hscore	45 (1-160); 25	45 (1-150); 22	35 (1-185); 23	30 (1-150); 11	
HercepTestTM 	0 (0-3); 24	0 (0-3); 21	0 (0-3); 21	0 (0-2); 8	
pHER2c Hscore	0 (0-30); 25	0 (0-40); 22	0 (0-50); 23	5 (0-40); 11	
pHER2m Hscore	50 (4-160); 25	65 (2-160); 22	45 (1-160); 23	40 (2-145); 11	
EGFRc Hscore	50 (5-125); 25	43 (5-150); 22	45 (5-110); 23	25 (10-130); 11	
EGFRm HScore	6 (1-45); 25	6 (1-70); 22	10 (1-25); 23	7 (2-25); 11	
pEGFRc HScore 	2 (1-120); 25	1 (1-25); 22	1 (1-40); 23	10 (1-55); 11	
pEGFRm HScore	3 (1-40); 25	2 (1-40); 22	1 (1-14); 23	7 (1-80); 11	
pMAPK HScore	60 (2-165); 25	38 (2-105); 22	25 (2-120); 23	50 (20-90); 11	
Ki67 % 	18 (1-60); 25	4 (1-32); 22	3 (1-45); 23	15 (1-40); 11	
Tumour cellularity %	35 (10-60); 25	30 (0-45); 23	20 (5-55); 24	35 (5-40); 11	
T1= pre-treatment; T2= 6 week fulvestrant treatment; T3= 6 month fulvestrant treatment; 
T4= fulvestrant progression samples in CB (clinical benefit) patient group; no.=  patient numbers; 
c= cytoplasmic; m= membrane. 


Supplementary Table S4: Median level of markers at each biopsy time-point in fulvestrant early progressors (EP) and continuing responders/late progressors (CONR/LP).
Markers 
	Median T1 
(Range); no.	Median T2 
(Range); no.	Median T3 
(Range); no.	
ER Hscore	130 (70-190); 11           130 (60-180); 12	80 (25-130); 7         
80 (0-140); 13	45 (5-130); 11              52.5 (2-135);10                                	
PR Hscore	50 (0-270); 11
55 (3-230); 12	15 (0-260); 8
30 (0-210); 13	1 (0-190); 9
17.5 (0-210); 8	
Bcl-2 %	75 (15-95); 11 
72.5 (50-90); 12	42.5 (10-80); 8 
80 (25-95); 13	42 (7-90); 11 
67.5 (10-90); 10	
pER HScore        	50 (4-80); 11
42.5 (10-140); 12	40 (5-115); 7
40 (7-120); 13	34 (5-85); 11
42.5 (7-120); 10	
HER2c Hscore	0 (0-60); 11                       0 (0-0); 12	0 (0-25); 7                 
0 (0-15); 13	0 (0-45); 11                       0 (0-20); 10	
HER2m Hscore	45 (1-160); 11
60 (10-110); 12	35 (1-150); 7           
54 (3-140); 13	35 (1-155); 11                 50 (15-120); 10	
HercepTestTM 	0 (0-1); 11
0 (0-1); 11	0 (0-2); 7
0 (0-1);12	0 (0-2); 10
0 (0-2); 9	
pHER2c Hscore	5 (0-30); 11
0 (0-5); 12	5 (0-20); 7
0 (0-40);13	0 (0-50); 11
0 (0-20); 10	
pHER2m Hscore	45 (4-160); 11
72.5 (19-150); 12	35 (2-140); 7
95 (3-160); 13	45 (1-160); 11
52.5 (13-135); 10	
EGFRc HScore	35 (10-125); 11
55 (5-110); 12	35 (5-115); 7
50 (5-140); 13	40 (10-70); 11
50 (5-105); 10	
EGFRm HScore	 4 (1-45); 11
8(1-35); 12	10 (1-25); 7
5 (1-25); 13	9 (1-25); 11
 8.5 (1-25); 10	
pEGFRc HScore 	5 (1-120); 11
1 (1-11); 12	1 (1-20); 7
1(1-25); 13	1 (1-20); 11
5 (1-40); 10	
pEGFRm HScore	3 (1-23); 11
1 (1-40); 12	5 (1-13); 7
1 (1-40); 13	1 (1-10); 11
1 (1-5); 10	
pMAPK HScore	60 (3-125); 11
65 (5-165); 12	35 (15-55); 7
40 (2-105); 13	35 (6-120); 11
17.5 (6-120); 10	
Ki67 % 	30 (1-60); 11 
11.5 (1-30); 12	5 (1-20); 7 
3 (1-12); 13	10 (1-45); 11
1(1-5); 10	
Tumour cellularity %	35 (10-50); 11
38 (15-60); 12	25 (0-45); 8
30 (5-45); 13	20 (5-40); 11
15 (5-40); 11	
T1= pre-treatment; T2= 6 week fulvestrant treatment; T3= 6 month fulvestrant treatment; 
no.=  patient numbers; c= cytoplasmic; m= membrane in the EP (early progressor) patient group. Equivalent time-point data for the CONR/LP (continuing responder/late progressor) patient group are indicated in italics.
Supplementary Table S5: Association between T1 (pre-treatment) Ki67 status and duration of response (DoR) on fulvestrant using Cox Proportional Hazards model. 

Covariate 		Relative risk 	        95% confidence interval        p value
T1 Ki67   		6.608 			1.521 - 28.711			0.0121
T1 pHER2c		3.010			0.834 - 10.863			0.092
Tumour grade:									0.217
Grade 2 vs. 1		0.455			0.062 - 3.357			0.440
Grade 3 vs. 1		1.510 			0.113 - 20.128			0.755
Site of disease 		3.763 			1.285 - 11.018 		0.0161

1 Statistically significant (p< 0.05).

(T1 Ki67 and disease site are significant independent predictors of fulvestrant DoR. Increased risk of relapse is associated with higher T1 Ki67 staining (>18% positivity), or for systemically-advanced (metastatic) disease versus locally-advanced disease at baseline).
.
