# Supplementary material for: Biological effects of fulvestrant on estrogen receptor positive human breast cancer: short, medium and long‐term effects based on sequential biopsies
Source: Int J Cancer. 2015 Jul 30;138(1):146–59. doi: 10.1002/ijc.29682 (PMC4879515; doi:10.1002/ijc.29682)
Supplement: Supplementary file 3 — Supporting Information LAB methods [file IJC-138-146-s003.rtf]

SuppLEMENTARY Information for IMMUNOHISTOCHEMICAL STAINING AND ASSESSMENT
Biological effects of fulvestrant on estrogen receptor positive human breast cancer: short, medium and long-term effects based on sequential biopsies 
Agrawal A, Robertson JFR, Cheung KL, Gutteridge E, Ellis IO, Nicholson RI, Gee JMW.

I. General methods for all assays
II. Specific methods for individual markers    
III. Staining assessment
IV. Methodology references

I. General methods for all assays
The antibodies employed for immunohistochemistry (IHC) in this study have previously been demonstrated extensively to be specific by Western Blotting, including employment of pharmacological challenge with specific signal transduction inhibitors in vitro [1-3]. Importantly, phosphorylation sites chosen for analysis in the present study have also previously been reported to be key residues recruited during activation of the signaling molecule under test. Furthermore, the IHC assay procedures employed have previously been demonstrated to be sensitive and specific using in vitro and/or clinical cancer preparations [4, 5],with paraffin-embedded clinical breast cancer material employed for optimisation and validation of all these assays (including antigen retrieval). Assays for total ER, PR and Ki67 have also previously been utilized in short-term studies with fulvestrant [6].
Effective, equivalent and prompt handling of all core biopsy specimens comprising this study by a trained research laboratory officer aimed to maximise retention and stability of antigens in the tumour specimens, as did utilizing fresh sections from each tumour block and ensuring all samples from a given patient were cut at the same time.  For all assays in this study, matched sequential breast cancer core biopsy samples for each anonymised patient were always ran in assays together to reduce impact of any inter-assay variability. Assays always included a paraffin-embedded breast cancer slide of known marker positivity for quality-control purposes. 
Paraffin sections from each formalin-fixed biopsy (cut at 3 microns onto charged slides) were 
de-waxed and re-hydrated using sequential solutions of xylene, ethanol (decreasing concentrations), distilled water and phosphate buffered saline (PBS 0.01M pH 7). Hydrogen peroxide (3%) was used to block endogenous peroxidases followed by washing with distilled water. Sections were retrieved by immersing in buffers of appropriate pH and either heat or enzymatically-treated as appropriate followed by IHC assay as detailed in Sections IIa-j below. All antibody incubations were carried out in a humidity chamber. Buffer washes (all using PBS 0.01M [pH 7] or Tween [0.02%]/0.01M PBS [pH 7]) were performed between all primary antibody and subsequent immunodetection steps. Sections were finally exposed to 
3-3 diaminobenzidine tetrahydrochloride (DAB) chromogen (Dako, Ely, UK), followed by counterstaining, in general using 0.5% methyl green. Slides were dehydrated, coverslipped and permanently mounted, drying prior to staining assessment.

II. Specific methods for individual markers    
a)	ER 
Antigen retrieval was performed by pressure cooking sections in 0.01M sodium citrate (pH 6) for 2 minutes. After blocking for non-specific binding (using 20% normal human serum [NHS]), primary antibody (ER clone 6F11 mouse monoclonal antibody at 1/200 NovoCastra, Milton Keynes, UK) was applied for 60 minutes at room temperature. The antibody was prepared in 
a 0.01M PBS (pH 7) buffer that was also used for all further IHC assays. Detection employed Dako EnVision+™ peroxidase-labeled polymer anti-mouse (Dako, Ely, UK) for 75 minutes at room temperature followed by the chromogen and counterstaining steps.
b)	pER 
Antigen retrieval was performed by pressure cooking sections in 0.01M sodium citrate (pH 6) for 2 minutes. After blocking for non-specific binding (using 0.02% Tween/0.01M PBS) for 5 minutes, primary antibody (serine 118-phosphorylated ER mouse monoclonal antibody 1/1700 in 0.01M PBS; Cell Signaling, Hitchin, UK) was applied overnight at room temperature. Detection was carried out using Dako EnVision+™ peroxidase-labeled polymer anti-mouse for 2 hours at room temperature followed by the chromogen and counterstaining steps.
c)	Bcl-2
Antigen retrieval was performed by microwaving sections in 0.01M citric acid (pH 6) for 30 minutes at half-power in a Proline Micro Chef ST44 microwave (full-power setting: 960W; half-power setting: 560W). After blocking for non-specific binding (using 5% normal rabbit serum [NRS] and 5% NHS in 0.1% BSA/0.01M PBS) for 10 minutes, primary antibody (Dako Bcl-2 mouse monoclonal antibody M0887 at 1/30 in 0.1% BSA/0.01M PBS) was applied overnight at room temperature. Detection was carried out using Dako EnVision+™ peroxidase-labeled polymer anti-mouse for 2 hours followed by the chromogen and counterstaining steps.
d)	PR 
Antigen retrieval was performed by microwaving sections in 0.01M citrate buffer (pH 6) for 10 minutes at 800W followed by a further 10 minutes at half-power. After blocking for non-specific binding using normal swine serum (NSS), primary antibody (Dako mouse monoclonal antibody clone PR 636) used at 1/200 in NSS/Tris buffered saline [NSS/TBS; 0.1M pH 7.5]) was applied for 45 minutes at room temperature. Secondary biotinylated anti-mouse antibody (Dako, at 1/100 dilution in NSS/TBS) was applied for 30 minutes. Detection was carried out using streptavidin-biotin/horseradish peroxidase complex (Dako, diluted 1/100) for 55 minutes at room temperature followed by the chromogen and counterstaining steps.
e)	Total HER2 
i.	HercepTest™
Sections were incubated at 60°C for 10 minutes (to aid section adherence to the slides), prior to dewaxing in xylene for 10 minutes. Sections were then re-hydrated through decreasing grades of industrial methylated spirits (IMS) and placed in running tap water. Antigen retrieval was performed strictly according to the HercepTest™ kit protocol (Dako Ltd, Denmark). This involved incubation of sections in kit epitope retrieval solution for 40 minutes in a water bath at 95-99°C, then cooling at room temperature for 20 minutes. IHC staining for HER2 was performed on the Dako TechMate™ automated stainer using kit K5206 and following the protocol supplied. The staining method involved blocking endogenous peroxidase activity, incubation in the primary HER2 antibody, incubation in visualization reagent, incubation with  DAB chromogen, and counterstaining with haematoxylin (with wash steps in buffer between each step). Sections were then de-hydrated through increasing grades of IMS, cleared in xylene and mounted.
ii.	HER2
Along with the HercepTest™, a further HER2 assay with increased sensitivity for very low levels of cytoplasmic HER2 (in addition to detecting HER2 plasma membrane staining) was used. Antigen retrieval was performed by microwaving sections in 0.01M citric acid (pH 6) for 30 minutes at half-power (560W). After blocking for non-specific binding (using 5% normal goat serum [NGS]/5% NHS in 0.01M PBS for 15 minutes), primary antibody (Dako A0485 polyclonal rabbit anti-human HER2 at 1/250 in 5% NGS/5% NHS in 0.01M PBS) was applied for 2 hours at room temperature. Detection was carried out using 1/50 goat anti-rabbit peroxidase-labeled IgG (Sigma, Gillingham, UK; A4914; prepared in 5% NGS/5% NHS in 0.01M PBS) for 60 minutes at room temperature followed by the chromogen and counterstaining steps.
f)	pHER2
Antigen retrieval was performed by pressure cooking sections in 0.01M sodium citrate (pH 6) for 2 minutes. After blocking for non-specific binding (using 1% BSA/0.01M PBS for 10 minutes), primary antibody (phosphorylated HER2 antibody at 1/250 to detect pY1248 HER2; Upstate, Watford, UK) was applied overnight at room temperature. Detection was carried out using Dako EnVision+™ peroxidase-labeled polymer anti-mouse for 2 hours at room temperature followed by the chromogen and counterstaining steps.
g)	EGFR
Antigen retrieval was performed by enzymatic digestion sections using 0.02% protease (Sigma P6911) in 0.01M PBS at 37oC for 20 minutes. After blocking for non-specific binding (using 5% BSA/0.01M PBS for 10 minutes), primary antibody (mouse monoclonal EGFR antibody at 1/30 in 0.01M PBS; NeoMarkers, Loughborough, UK) was applied overnight at room temperature. Secondary antibody (“Mouse Link”; comprising biotinylated anti-mouse immunoglobulins at 1/40 in 1% BSA/0.01M PBS; Biogenex, Longfield, UK) was applied for 60 minutes. Detection was carried out using Biogenex “Label” (streptavidin-peroxidase) at 1/40 in 1% BSA/0.01M PBS for 60 minutes followed by the chromogen and counterstaining steps.
h)	pEGFR
Antigen retrieval was performed by microwaving sections in 0.01M EDTA (pH 8) for 1 minute at full-power (960W) followed by 9 minutes at half-power (560W), holding the slides in the EDTA buffer for a further 20 minutes. After blocking for non-specific binding (using 0.02% Tween/0.01M PBS for 5 minutes), primary antibody (rabbit anti-phosphorylated EGFR polyclonal antibody at 1/80 in 1% BSA/0.01M PBS to detect pY845 EGFR; Biosource, Paisley, UK) was applied overnight at room temperature. Detection was carried out using Dako EnVision+™ peroxidase-labeled polymer anti-rabbit for 2 hours at room temperature followed by the chromogen and counterstaining steps.
i)	pMAPK
Antigen retrieval was performed by microwaving sections in 0.01M citric acid (pH 6) for 30 minutes at half-power (560W). After blocking for non-specific binding (using 20% NHS/0.01M PBS), primary antibody (rabbit polyclonal antibody at 1/20 in 20% NHS/0.01M PBS to detect p42/p44 Thr202/Tyr204 ERK1 and ERK2 MAP kinase phosphorylation; Cell Signaling, Hitchin, UK) was applied overnight at room temperature. A secondary antibody (Biogenex “Multi Link” comprising biotinylated immunoglobulins for mouse, rabbit, guinea pig or rat antibodies, 1/100 in 1% BSA/0.01M PBS) was applied for 60 minutes. Detection was carried out using Biogenex “Label” (streptavidin-peroxidase) at 1/100 in 1% BSA/0.01M PBS for 60 minutes followed by the chromogen and counterstaining steps.
j)	Ki67 proliferation staining 
Antigen retrieval was performed by microwaving sections in 0.01M citric acid (pH 6) for 30 minutes at half-power (560W). After blocking for non-specific binding (using 10% NRS), primary antibody (Dako Ki67 antigen clone MIB1 M7240, at 1/50 in 0.1% BSA/0.01M PBS) was applied for 2 hours at room temperature. Detection was carried out using a secondary antibody (Biogenex “Mouse Link” comprising biotinylated anti-mouse immunoglobulins at 1/40 in 1% BSA/0.01M PBS) for 25 minutes followed by application of Biogenex “Label” (streptavidin-peroxidase) at 1/40 in 1% BSA/0.01M PBS for 25 minutes and the chromogen and counterstaining steps.


III. Staining Assessment
IHC staining assessment was completed by two experienced observers who did not have knowledge of patients' clinical data, examining all immunostained samples for each patient. Tumour epithelial immunopositivity comprised a brown signal against green or blue counterstained negative cells. Any stromal cell immunostaining or normal/benign breast structures were not considered in this assessment. Samples with unacceptable background staining, insufficient tumour material (<100 tumour cells), or very poor or equivocal histological structure were eliminated. Positive control sections were monitored for each marker to ensure optimal assay performance, while negative controls were checked for background staining. Immunostaining in the following cellular localisation was evaluated:
Nuclear: ER, pER, PR, Ki67; pMAPK
Cytoplasmic: Bcl-2
Plasma membrane and cytoplasmic: EGFR, pEGFR, HER2, pHER2
Plasma Membrane score: HercepTestTM
A light microscope (BH2; Olympus™ Optical Company, Germany) with dual-viewing attachment was used for assessment. Overall examination of tumour epithelial immunostaining was first performed at an ocular magnification of x10 to eliminate any associated normal/benign structures and to locate representative areas of tumour for further analysis. These tumour areas were then viewed at x40 for more detailed tumour epithelial cell immunostaining assessment. The two experienced observers simultaneously assessed percentage tumour epithelial staining within five intensity categories (i.e. negative, very weak +/-, weak +, moderate ++ and strong +++). These data were used to derive a consensus HScore value for every specimen. This well-established immunostaining index on a 0-300 scale was derived using the following accepted formula: HScore = (0.5 x % very weak staining cells) + (1 × % weak staining cells) + (2 × % moderate staining cells ) + (3 × % strong staining cells). HER2 assessment for the HercepTest™ was based on the strength of plasma membrane immunostaining of 10% or more of cells (no staining or <10% = score 0; faint/partial staining = score 1+; weak/moderate staining = score 2+; strong/complete staining = score 3+). Samples with scores of 3+ were deemed HER2 protein overexpressors by HercepTestTM. For Ki67 immunostaining, counting of percentage positivity determined proliferating cells vs. negative (G0) cells. Percentage positivity was also evaluated for Bcl-2 staining. Up to five representative fields at × 20 magnification were chosen from each section and then examined in detail at × 40 magnification, from which the average positivity was determined for each specimen.
Methodology references

1.	Gee JM, Howell A, Gullick WJ et al. Consensus statement. Workshop on therapeutic resistance in breast cancer: impact of growth factor signalling pathways and implications for future treatment. Endocr Relat Cancer 2005; 12 Suppl 1: S1-7.

2.	Knowlden JM, Hutcheson IR, Barrow D et al. Insulin-like growth factor-I receptor signaling in tamoxifen-resistant breast cancer: a supporting role to the epidermal growth factor receptor. Endocrinology 2005; 146: 4609-18.

3.	Knowlden JM, Hutcheson IR, Jones HE et al. Elevated Levels of Epidermal Growth Factor Receptor/c-erbB2 Heterodimers Mediate an Autocrine Growth Regulatory Pathway in Tamoxifen-Resistant MCF-7 Cells. Endocrinology 2003; 144: 1032-44.

4.	Gee JM, Harper ME, Hutcheson IR et al. The antiepidermal growth factor receptor agent gefitinib (ZD1839/Iressa) improves antihormone response and prevents development of resistance in breast cancer in vitro. Endocrinology 2003; 144: 5105-17.

5.	Gutteridge E, Agrawal A, Nicholson R et al. The effects of gefitinib in tamoxifen-resistant and hormone-insensitive breast cancer: a phase II study. Int J Cancer 2010; 126: 1806-16.

6.	Robertson JF, Nicholson RI, Bundred NJ et al. Comparison of the short-term biological effects of 7alpha-[9-(4,4,5,5,5-pentafluoropentylsulfinyl)-nonyl]estra-1,3,5, (10)-triene-3,17beta-diol (Faslodex) versus tamoxifen in postmenopausal women with primary breast cancer. Cancer Res 2001; 61: 6739-46.


.
