# Supplementary material for: Biological effects of fulvestrant on estrogen receptor positive human breast cancer: short, medium and long‐term effects based on sequential biopsies
Source: Int J Cancer. 2015 Jul 30;138(1):146–59. doi: 10.1002/ijc.29682 (PMC4879515; doi:10.1002/ijc.29682)
Supplement: Supplementary file 1 — Supporting Information Figure s1 [file IJC-138-146-s001.rtf]

SUPPLEMENTARY FIGURE S1. Biological effects of fulvestrant on estrogen receptor positive human breast cancer: short, medium and long-term effects based on sequential biopsies. 
Agrawal A, Robertson JFR, Cheung KL, Gutteridge E, Ellis IO, Nicholson RI, Gee JMW.

Immunohistochemistry (IHC) in sequential clinical breast cancer samples taken prior to (T1) and during (6 weeks: T2; 6 months: T3) fulvestrant treatment and at subsequent progression (T4) showing (a) ER; (b) PR; (c) Bcl-2; (d) pER (serine 118 phosphorylation); (e) and (f) HER2; (g) and (h) pHER2; (i) EGFR; (j) pEGFR; (k) pMAPK and (l) Ki67 staining. Images (f) and (h) show induction of HER2 expression and activity respectively in CB (clinical benefit) patients at T3. Original magnification x 20 for (a, e, g) and x 40 for (b, c, d, f, h, i, j, k, l) with scale bars= 40 and 20μm respectively.

S1 (a): ER sequential sample IHC (case no. 25).  ER at T1

 ER at T2

 ER at T3 

 ER at T4 


 S1 (b): PR sequential sample IHC (case no. 30)      

  PR at T1 

  PR at T2 

  PR at T3 

  PR at T4 


 S1 (c): Bcl-2 sequential sample IHC (case no. 25)

  Bcl-2 at T1

  Bcl-2 at T2

  Bcl-2 at T3 

  Bcl-2 at T4 


      S1 (d): pER (serine 118 phosphorylation) sequential sample IHC (case no. 16) 

       pER at T1 

       pER at T2 

       pER at T3
 
       pER atT4 


S1 (e): HER2 sequential sample IHC (case no. 25) 

 HER2 at T1 

 HER2 at T2 

 HER2 at T3 

 HER2 at T4 


S1 (f): Sequential sample IHC showing T3 increase in HER2 (case no. 21) 

 
 HER2 at T1 

 HER2 at T2 

 HER2 at T3 


      
      S1 (g): pHER2 sequential sample IHC (case no. 25)

 pHER2 at T1 

 pHER2 at T2 

 pHER2 at T3 

 pHER2 at T4 


S1 (h): Sequential sample IHC showing T3 increase in pHER2 (case no. 14) 

 
 pHER2 at T1 
 
 pHER2 at T2 

 pHER2 at T3 


S1 (i): EGFR sequential sample IHC (case no. 30) 

    EGFR at T1
 
 EGFR at T2 

EGFR at T3 

 EGFR at T4 


 S1 (j): pEGFR sequential sample IHC (case no. 11)

 pEGFR at T1 

 pEGFR at T2 

 pEGFR at T3 

 pEGFR at T4 


S1 (k): pMAPK sequential sample IHC (case no. 25)

 pMAPK at T1 


 pMAPK at T2 

 pMAPK at T3

 pMAPK at T4


S1 (l): Ki67 sequential sample IHC (case no. 11)

 Ki67 at T1 

 Ki67 at T2 

 Ki67 at T3

 Ki67 at T4 
.
